# COVID-19 severity and mortality in patients with CLL: an update of the international ERIC and Campus CLL study

**DOI:** 10.1038/s41375-021-01450-8

**Published:** 2021-11-01

**Authors:** Thomas Chatzikonstantinou, Anargyros Kapetanakis, Lydia Scarfò, Georgios Karakatsoulis, David Allsup, Alejandro Alonso Cabrero, Martin Andres, Darko Antic, Mónica Baile, Panagiotis Baliakas, Dominique Bron, Antonella Capasso, Sofia Chatzileontiadou, Raul Cordoba, Juan-Gonzalo Correa, Carolina Cuéllar-García, Lorenzo De Paoli, Maria Rosaria De Paolis, Giovanni Del Poeta, Christos Demosthenous, Maria Dimou, David Donaldson, Michael Doubek, Maria Efstathopoulou, Barbara Eichhorst, Shaimaa El-Ashwah, Alicia Enrico, Blanca Espinet, Lucia Farina, Angela Ferrari, Myriam Foglietta, Henrik Frederiksen, Moritz Fürstenau, José A. García-Marco, Rocío García-Serra, Massimo Gentile, Eva Gimeno, Andreas Glenthøj, Maria Gomes da Silva, Odit Gutwein, Yervand K. Hakobyan, Yair Herishanu, José Ángel Hernández-Rivas, Tobias Herold, Idanna Innocenti, Gilad Itchaki, Ozren Jaksic, Ann Janssens, Оlga B. Kalashnikova, Elżbieta Kalicińska, Linda Katharina Karlsson, Arnon P. Kater, Sabina Kersting, Jorge Labrador, Deepesh Lad, Luca Laurenti, Mark-David Levin, Enrico Lista, Alberto Lopez-Garcia, Lara Malerba, Roberto Marasca, Monia Marchetti, Juan Marquet, Mattias Mattsson, Francesca R. Mauro, Ivana Milosevic, Fatima Mirás, Marta Morawska, Marina Motta, Talha Munir, Roberta Murru, Carsten U. Niemann, Raquel Nunes Rodrigues, Jacopo Olivieri, Lorella Orsucci, Maria Papaioannou, Miguel Arturo Pavlovsky, Inga Piskunova, Viola Maria Popov, Francesca Maria Quaglia, Giulia Quaresmini, Kristian Qvist, Gianluigi Reda, Gian Matteo Rigolin, Rosa Ruchlemer, Gevorg Saghumyan, Amit Shrestha, Martin Šimkovič, Martin Špaček, Paolo Sportoletti, Oana Stanca, Niki Stavroyianni, Tamar Tadmor, Doreen Te Raa, Sanne H. Tonino, Livio Trentin, Ellen Van Der Spek, Michel van Gelder, Roel van Kampen, Marzia Varettoni, Andrea Visentin, Candida Vitale, Ewa Wasik-Szczepanek, Tomasz Wróbel, Lucrecia Yáñez San Segundo, Mohamed Yassin, Marta Coscia, Alessandro Rambaldi, Emili Montserrat, Robin Foà, Antonio Cuneo, Kostas Stamatopoulos, Paolo Ghia

**Affiliations:** 1 Hematology Department and HCT Unit, G. Papanicolaou Hospital, Thessaloniki, Greece; 2 Institute of Applied Biosciences, Centre for Research and Technology Hellas, Thessaloniki, Greece; 3 Università Vita-Salute San Raffaele and IRCC Ospedale San Raffaele, Milan, Italy; 4 Department of Mathematics, University of Ioannina, Ioannina, Greece; 5 Centre for Atherothrombosis and Metabolic Disease, Hull York Medical School, Hull, UK; 6 Spanish Society of Haematology and hemotherapy (SEHH: Sociedad Española de Hematología y hemoterapia), Madrid, Spain; 7 Hematology Department, Hospital Universitario de La Princesa, Madrid, Spain; 8 Department of Hematology and Central Hematology Laboratory, Inselspital, Bern University Hospital, University of Bern, Bern, Switzerland; 9 University Clinical Center of Serbia, Belgrade, Serbia; 10 School of Medicine, University of Belgrade, Belgrade, Serbia; 11 Hospital Clinico Universitario de Salamanca (CAUSA / IBSAL), Salamanca, Spain; 12 Department of Immunology, Genetics and Pathology, Science for Life Laboratory, Uppsala University, Uppsala, Sweden; 13 Department of Clinical Genetics, Uppsala University Hospital, Uppsala, Sweden; 14 Inst J Bordet (ULB), Brussels, Belgium; 15 IRCSS Ospedale San Raffaele, Milan, Italy; 16 Hematology Unit, 1st Dept of Internal Medicine, AUTH, AHEPA Hospital, Thessaloniki, Greece; 17 Department of Hematology, Health Research Institute IIS-FJD, Fundacion Jimenez Diaz University Hospital, Madrid, Spain; 18 Hospital Clínic of Barcelona, Barcelona, Spain; 19 Hematology Unit Terrassa Hospital, Terrasa, Spain; 20 Division of Hematology, Department of Translational Medicine, Università del Piemonte Orientale Amedeo Avogadro, Azienda Ospedaliero-Universitaria Maggiore della Carità Novara, Novara, Italy; 21 UOC Ematologia PO Vito Fazzi Lecce, Lecce, Italy; 22 Department of Biomedicine and Prevention Hematology, University Tor Vergata, Rome, Italy; 23 1st Internal Medicine Department, Propaedeutic, Hematology Clinical Trial Unit, National and Kapodistrian University of Athens, Athens, Greece; 24 Belfast City Hospital, Belfast, Northern Ireland; 25 Department of Internal Medicine – Hematology and Oncology, University Hospital, Brno; Department of Medical Genetics and Genomics, Faculty of Medicine, Masaryk University, Brno, Czech Republic; 26 Department of Haematology Athens Medical Center-Psychikon Branch, Athens, Greece; 27 Department I of Internal Medicine, Center for Integrated Oncology Aachen Bonn Cologne Duesseldorf (CIO ABCD), University Hospital Cologne, University of Cologne, Cologne, Germany; 28 Clinical Hematology Unit, Oncology Center, Faculty of Medicine, Mansoura University, Mansoura, 35516 Egypt; 29 Hospital Italiano La Plata, Buenos Aires, Argentina; 30 Pathology Service, Hospital del Mar, Barcelona, Spain; 31 Hematology, Fondazione IRCCS Istituto Nazionale Tumori, Milan, Italy; 32 Hematology Unit, Azienda Unità Sanitaria Locale – IRCCS, Reggio Emilia, Italy; 33 Division of Hematology, AO S. Croce e Carle, Cuneo, Italy; 34 Department of Hematology, Odense University Hospital, Odense, Denmark; 35 Hematology Department, Hospital Universitario Puerta de Hierro-Majadahonda, Madrid, Spain; 36 Department of Hematology, Hospital General Universitario, Valencia, Spain; 37 Fundación de Investigación del Hospital General Universitario, Valencia, Spain; 38 Hematology Unit AO Cosenza, Cosenza, Italy; 39 Department of Hematology, Hospital del Mar, Barcelona, Spain; 40 Department of Hematology, Rigshospitalet, Copenhagen University Hospital, Copenhagen, Denmark; 41 Hematology Department, Portuguese Institute of Oncology, Lisbon, Portugal; 42 Department of Hematology, Shamir Medical Center, Zerifin, Israel; 43 Sackler Faculty of Medicine, Tel Aviv University, Tel Aviv, Israel; 44 Hematology Center after Prof. Yeolyan MH RA, Yerevan, Armenia; 45 Department of Hematology, Tel Aviv Sourasky Medical Center and Sackler School of Medicine, Tel Aviv University, Tel Aviv, Israel; 46 Hematology Department Infanta Leonor University Hospital, Madrid, Spain; 47 Laboratory for Leukemia Diagnostics, Department of Medicine III, University Hospital, LMU Munich, Munich, Germany; 48 Dipartimento di Diagnostica per Immagini, Radioterapia Oncologica ed Ematologia, Fondazione Policlinico Universitario Agostino Gemelli IRCCS, Rome, Italy; 49 Rabin Medical Center, Petah Tikva, and the Sackler School of Medicine, Tel-Aviv University, Tel-Aviv, Israel; 50 Department of Hematology, University hospital Dubrava, Zagreb, Croatia; 51 Department of Hematology, Universitaire ziekenhuizen Leuven, Herestraat 49, 3000 Leuven, Belgium; 52 Federal State Budgetary Educational Institution of Higher Education Academician I.P. Pavlov First St. Petersburg State Medical University of the Ministry of Healthcare of Russian Federation, St. Petersburg, Russia; 53 Department and Clinic of Hematology, Blood Neoplasms and Bone Marrow Transplantation Wroclaw Medical University, Pasteura Street 4, 50-367 Wroclaw, Poland; 54 Dept of Hematology, Cancer Center Amsterdam, Amsterdam University Medical Centers, University of Amsterdam, Amsterdam, the Netherlands; 55 Department of Hematology, Haga Teaching Hospital, The Hague, The Netherlands; 56 Hematology Department, Unit Research, Complejo Asistencial Universitario de Burgos, Burgos, Spain; 57 Department of Internal Medicine, Postgraduate Institute of Medical Education and Research, Chandigarh, India; 58 Sezione di Ematologia, Dipartimento di Scienze Radiologiche ed Ematologiche, Università Cattolica del Sacro Cuore, Rome, Italy; 59 Department of Internal Medicine, Albert Schweitzer hospital, Dordrecht, the Netherlands; 60 Department of Hematology, Santa Chiara Hospital, Trento, Italy; 61 Hematology and Stem Cell Transplant Center Marche Nord Hospital, Pesaro, Italy; 62 Dept of Medical Sciences, Section of Hematology, University of Modena and Reggio E., Modena, Italy; 63 Hematology Unit and BM Transplant Center, AO SS Antonio e Biagio e Cesare Arrigo, Alessandria, Italy; 64 Hematology Department, Ramón y Cajal University Hospital, Madrid, Spain; 65 Department of Hematology, Uppsala University Hospital, Uppsala, Sweden; 66 Hematology, Department of Translational and Precision Medicine, Sapienza University, Rome, Italy; 67 Faculty of Medicine, Clinical Centre of Vojvodina, University of Novi Sad, Novi Sad, Serbia; 68 Hematology Department Hospital Universitario 12 de Octubre, Madrid, Spain; 69 Experimental Hematooncology Department, Medical University of Lublin, Lublin, Poland; 70 Hematology Department, St. John’s Cancer Center, Lublin, Poland; 71 S.C. Ematologia ASST Spedali Civili Brescia, Brescia, Italy; 72 Consultant Haematologist, St James’s Hospital, Leeds, LS9 7TF UK; 73 Hematology and Stem Cell Transplantation Unit, Ospedale Oncologico A. Businco, ARNAS “G. Brotzu”, Cagliari, Italy; 74 Hematology Clinic, ASUFC, Udine, Italy; 75 S.C. Ematologia, Città della Salute e della Scienza di Torino, Turin, Italy; 76 FUNDALEU, Clinical research center Buenos Aires, Buenos Aires, Argentina; 77 Consultative Hematology Department with a Day Hospital for Intensive High-Dose Chemotherapy, National Research Center for Hematology, Moscow, Russia; 78 HematologyDepartment, Colentina Clinical Hospital, Bucharest, Romania; 79 Department of Medicine, Section of Hematology, University of Verona, Verona, Italy; 80 Department of Oncology and Hematology Azienda Socio Sanitaria Territoriale Papa Giovanni XXIII Bergamo, Bergamo, Italy; 81 Hematologic Section, Dept. of Internal Medicine, Hospital Union West, Herning, Denmark; 82 Hematology Unit, Foundation IRCCS Ca’ Granda Ospedale Maggiore Policlinico of Milan, Milan, Italy; 83 St. Anna University Hospital, Ferrara, Italy; 84 Department of Hematology, Shaare-Zedek Medical Center, affiliated with the Hebrew University Medical School, Jerusalem, Israel; 85 Hematology Unit, Nepal Cancer Hospital & Research Centre, Lalitpur, Nepal; 86 4th Department of Internal Medicine – Haematology, Faculty of Medicine in Hradec Králové, University Hospital and Charles University in Prague, Hradec Kralove, Czech Republic; 87 1st Department of Medicine - Hematology, First Faculty of Medicine, Charles University and General Hospital in Prague, Prague, Czech Republic; 88 Department of Medicine and Surgery, Institute of Hematology and Center for Hemato-Oncological Research, University of Perugia, Perugia, Italy; 89 “Carol Davila” University of Medicine and Pharmacy - Hematology Department from Coltea Clinical Hospital, Bucharest, Romania; 90 Hematology, Bnai-Zion Medical Center, Haifa, Israel; 91 Department of hematology, Gelderse Vallei Ede, Ede, the Netherlands; 92 Dept of Hematology, Lymmcare, Cancer Center Amsterdam, Amsterdam University Medical Centers, University of Amsterdam, Amsterdam, the Netherlands; 93 Hematology and Clinical Immunology Unit, Department of Medicine, University of Padova, Padova, Italy; 94 Department of Internal Medicine, Rijnstate Hospital, Arnhem, the Netherlands; 95 Dept Internal Medicine, Maastricht University Medical Center, Maastricht, the Netherlands; 96 Zuyderland Medical Center, Sittard, the Netherlands; 97 Division of Hematology Fondazione IRCCS Policlinico San Matteo, Pavia, Italy; 98 Division of Hematology, A.O.U. Città della Salute e della Scienza di Torino and Department of Molecular Biotechnology and Health Sciences, University of Turin, Turin, Italy; 99 Dept. Hematooncology and Bone Marrow Transplantation Medical University in Lublin, Lublin, Poland; 100 Hematology Department, University Hospital and Research Institute of Marqués de Valdecilla (IDIVAL), Santander, Spain; 101 Hematology Section, Department of Medical Oncology, National Center for Cancer Care and Research, Doha, Qatar; 102 Department of Oncology and Hematology, University of Milan, Milan, Italy

**Keywords:** Chronic lymphocytic leukaemia, Epidemiology

## Abstract

Patients with chronic lymphocytic leukemia (CLL) may be more susceptible to Coronavirus disease 2019 (COVID-19) due to age, disease, and treatment-related immunosuppression. We aimed to assess risk factors of outcome and elucidate the impact of CLL-directed treatments on the course of COVID-19. We conducted a retrospective, international study, collectively including 941 patients with CLL and confirmed COVID-19. Data from the beginning of the pandemic until March 16, 2021, were collected from 91 centers. The risk factors of case fatality rate (CFR), disease severity, and overall survival (OS) were investigated. OS analysis was restricted to patients with severe COVID-19 (definition: hospitalization with need of oxygen or admission into an intensive care unit). CFR in patients with severe COVID-19 was 38.4%. OS was inferior for patients in all treatment categories compared to untreated (*p* < 0.001). Untreated patients had a lower risk of death (HR = 0.54, 95% CI:0.41–0.72). The risk of death was higher for older patients and those suffering from cardiac failure (HR = 1.03, 95% CI:1.02–1.04; HR = 1.79, 95% CI:1.04–3.07, respectively). Age, CLL-directed treatment, and cardiac failure were significant risk factors of OS. Untreated patients had a better chance of survival than those on treatment or recently treated.

## Introduction

The Coronavirus disease 2019 (COVID-19) has cost the lives of more than 3 million people worldwide, since the first cases were reported in December 2019 in Wuhan, China [1]. While the severity of the disease can vary greatly between infected individuals, older age, and specific comorbidities confer a worse prognosis. Obesity, diabetes mellitus, cardiovascular, respiratory diseases, and cancer are associated with poor outcome [2, 3].

Patients with hematological malignancies often experience severe disease and have high case fatality (CFR) and mortality rates. A meta-analysis of 3240 adult patients with a hematological malignancy reported a risk of death of 34% [4]. Moreover, in a study of 740 patients with hematological malignancies from Turkey the CFR was significantly higher when compared with age, sex, and comorbidity-matched controls (13.8% vs 6.8%) [5]. In a similar patient group from Italy, Passamonti et al. reported a standardized mortality ratio of 2.04 (95% CI 1.77–2.34) compared with the Italian general population with COVID-19 [6].

Chronic lymphocytic leukemia (CLL) is the most frequent adult hematological malignancy in the West, affecting particularly the elderly, with a median age at diagnosis of 72 years [7]. CLL is characterized by an impaired immune capacity due to a profound immune dysregulation reflected by hypogammaglobulinemia, qualitative and quantitative B- and T-cell defects, CD4^+^ lymphopenia, innate immune dysfunction, and neutropenia [8]. Antileukemic treatments can further weaken the immune response to common pathogens, rendering patients more susceptible to infections [9]. CLL immune defects may hinder the host from effectively controlling SARS-CoV-2 replication. Conversely, while patients with CLL might struggle to eliminate the virus, they may be less prone to a cytokine-induced inflammatory hyperactivation that can lead to acute respiratory distress syndrome, multiorgan damage, and death [10].

Early in the pandemic, reports from hospitalized patients showed that those with hematological malignancies were more susceptible to severe infection from SARS-CoV-2 and had higher CFR compared to the general population [11, 12]. In those studies, CLL was underrepresented and the impact of specific antileukemic treatments was not assessed. In a COVID-19 and CLL-specific cohort of 46 Italian patients, the Campus CLL group reported a CFR of 30.4% [13]. Following this report, ERIC, the European Research Initiative on CLL, and Campus CLL reported data from 190 patients [14]. Among hospitalized patients, the CFR was 32.5%. Age and number of comorbidities did not impact on overall survival (OS); patients on recent or ongoing treatment were more likely to have milder COVID-19 compared to untreated patients; finally, patients treated with the Bruton’s tyrosine kinase inhibitor (BTKi) ibrutinib were less likely to be hospitalized, prompting us to suggest a possible protective effect.

Mato et al. reported the outcomes of 198 patients with COVID-19 and CLL mainly from the USA [15]. The overall CFR was 33%. No difference was observed between treated and untreated patients with regard to infection severity and mortality. Advanced age and specific comorbidities conferred a worse prognosis. BTKi did not show a protective effect; however, in most cases, they were held during the infection.

The ERIC and US cohorts were also analyzed together with a Spanish cohort in a joint effort. The CFR was 30–34%. Age was the only risk factor of fatality in both cohorts, while the effect of CLL treatment on OS was inconsistent across cohorts. None of the CLL-directed treatments affected OS [16].

As SARS-CoV-2 is still surging across the globe, we significantly expanded our retrospective international multicenter cohort of patients with CLL and COVID-19. We aimed at reassessing the risk factors for COVID-19-related fatality and elucidate further the impact of CLL-directed treatments.

## Methods

### Data collection

This is a retrospective international multicenter study of the ERIC and the Campus CLL. It represents a continuation of a previous study conducted during the first wave of the pandemic [14]. Investigators at each center updated the information of their previous cases and added any new patients with COVID-19 and CLL/small lymphocytic lymphoma (SLL) or high-count CLL-like monoclonal B-cell lymphocytosis (MBL), a pre-CLL condition, also characterized by inherent immune compromise [17]. The establishment of CLL diagnosis, treatment decisions, review of medical history, and assessment of patient status were performed by the local teams following international guidelines [18]. The study was approved by the institutional ethics committees and data were processed and treated lawfully and fairly in a transparent manner that ensured appropriate security of the personal data, abiding by the General Data Protection Regulation. Informed consent was obtained from all patients that survived the infection. Data collection took place between March 28 and May 22, 2020 (first study) and between December 01, 2020, and March 16, 2021 (current study). Investigators reported all their patients with CLL or related conditions (SLL or MBL) who were diagnosed with COVID-19 from the beginning of the pandemic until the completion of the collection.

Ninety-one institutions participated in the study providing information for a total of 1009 cases. After excluding cases with uncertain diagnosis (‘atypical CLL’) and those without a qRT-PCR positive test for SARS-CoV-2, we resulted in 941 patients to be studied, 190 of whom were included in the first study. The full list of participating countries is reported in Supplemental Table 1.

Data extracted from the medical records included: baseline demographics; date of CLL diagnosis; IGHV gene somatic hypermutation status; cytogenetic status for chromosomes 11q, 13q 17p, and 12 determined by fluorescence in situ hybridization (at last assessment); *TP53* gene mutation status assessed by Sanger sequencing or next-generation sequencing (at last assessment); CLL treatment status; presence, number, and type of comorbidities, date of COVID-19, COVID-19 symptoms, COVID-19 management, treatment, complications, and outcome.

OS was defined as the time from suspected COVID-19 to death or last follow-up date.

In keeping with international practice, patients were deemed to have COVID-19 if a qRT-PCR assay test from a throat or nose swab was positive for SARS-CoV-2.

Severe COVID-19 was defined as hospitalization and need of oxygen or admission into an intensive care unit; nonsevere/mild COVID-19 was defined as confinement at home or hospitalization without need of oxygen.

Patients diagnosed with COVID-19 from the start of the pandemic until June 30, 2020, were considered to belong to the 1st wave of SARS-CoV-2, while patients diagnosed from July 1st until the completion of data collection were designated to the 2nd wave. The cut-off was decided based on the disposition of cases in our cohort (Supplemental Fig. 1).

### Statistical analysis

Median (IQR) is used to describe numeric variables while frequencies and percentages are used for categorical. Both univariate and multivariate analyses were carried out, having COVID-19 disease severity, CFR, or OS as outcomes. As potential risk factors, we examined clinico-biological characteristics (age, comorbidities, gender, IGHV gene somatic hypermutation status, *TP53* aberrations), COVID-19 disease severity (where appropriate), CLL treatment status (at the time of COVID-19 and in the last 12 months), type of CLL treatment (at the time of COVID-19 and in the last 12 months), and measures taken for the management of COVID-19 (continued as planned, replaced with other, or stopped the treatment). Also, we compared CFR between the two waves. To this end, to reduce the impact of confounding factors, we firstly tested for differences of the above-mentioned risk factors between the two waves. The statistical analyses were carried out in either all patients or subsets of the dataset (only severe, only patients receiving BTKi at the time of COVID-19). In the analyses for CFR, patients who were still under medical care were excluded. The significance level was set to 5%. In post-hoc comparisons, the correction of Bonferroni was used.

For COVID-19 disease severity and CFR, *χ*^2^ test or Fisher’s exact test (when necessary) were used for univariate analyses, while logistic regression was used for the multivariate. When necessary, we performed bias reduction techniques to the logistic model estimates by adjusting Firth’s logistic regression. Furthermore, for the comparison of CFR between the two waves, age was used as a confounder, since statistically significant older patients were identified in the first wave. For the comparisons of the numeric risk factors between the two waves, independent samples *t*-tests (Student’s or Welch’s) were conducted. The homogeneity of variance between the two groups was examined through Levene’s test. For the categorical risk factors, *χ*^2^ test or Fisher’s exact test were used.

For OS, the log-rank test was used for the univariate analyses, and Cox regression was conducted for the multivariate.

For the multivariate analyses, we performed a two-level variable selection approach. At first, we obtained the statistically significant risk factors through univariate analyses and used them as risk factors for a multivariate model. We further explored the multivariate model by performing backward elimination using *p* value.

All statistical analyses were conducted using R 4.0.4. We used brglm package [19] for Firth’s logistic regression, survival [20], and survminer packages [21] for survival analysis and ggplot2 package [22] for data visualization (Kaplan–Meier curves and the line-plot).

## Results

### Patient characteristics

We included 941 patients with a median age of 69 years (IQR 61–77) at the time of SARS-CoV-2 infection. Most patients were diagnosed with CLL (887, 94.3%), while 38 and 16 patients were diagnosed with SLL and MBL, respectively. The majority (628/941, 66.7%) were male with a median number of comorbidities of 2. Baseline patient characteristics are shown in Table 1.

**Table 1:** Patient Characteristics.

Patient characteristics		Results	Missing
Age at COVID-19, years (median, IQR)		69 (61–77)	0 (0%)
Gender	Female	313 (33.3%)	0 (0%)
	Male	628 (66.7%)	
Diagnosis	CLL	887 (94.3%)	
	MBL	16 (1.7%)	
	SLL	38 (4%)	
Obesity (BMI > 30)		151 (17.3%)	70 (7.4%)
Smoking	Current smoker	73 (8.8%)	112 (11.9%)
	Ex-smoker	222 (26.8%)	
	Never	534 (64.4%)	
Hypogammaglobulinemia (IgG < 550 mg/dL)		352 (49.5%)	230 (24.4%)
CIRS score (median, range)		4 (0–32)	76 (8.1%)
N of comorbidities (median, range)		2 (0–11)	0 (0%)
Other respiratory		61 (6.5%)	4 (0.4%)
Asthma		21 (2.2%)	4 (0.4%)
COPD		61 (6.5%)	4 (0.4%)
Cardiac Failure		30 (3.2%)	4 (0.4%)
Arrythmias		87 (9.3%)	4 (0.4%)
Coronary artery disease		94 (10%)	4 (0.4%)
Other cardiovascular		83 (8.9%)	4 (0.4%)
Hypertension		440 (47%)	4 (0.4%)
Diabetes		173 (18.5%)	4 (0.4%)
Chronic renal disease		51 (5.4%)	4 (0.4%)
Other hematological malignancies		10 (1.1%)	4 (0.4%)
Other non-hematological malignancies (excluding skin)		75 (8%)	4 (0.4%)

*IQR* interquartile range, *CIRS* cumulative illness rating scale, *COPD* chronic obstructive pulmonary disease, *CLL* chronic lymphocytic leukemia, *SLL* small lymphocytic lymphoma, *MBL* monoclonal B lymphocytosis.

Regarding CLL history, 394 patients (41.9%) had never received treatment for CLL, while 547 (58.1%) had been treated with at least one line of treatment. Amongst treated patients, 320 (34%) were on treatment at the time of SARS-CoV-2 infection. The most common treatment category at the time of COVID-19 was BTKi monotherapy (56.3%), followed by venetoclax monotherapy (10.7%) and chemoimmunotherapy (9.1%). Detailed information about CLL-directed therapy is given in Table 2.

**Table 2:** CLL-directed treatment.

Treatment	Category	Number	Percentage	Missing
CLL treatment status	Treated	547	58.1%	0 (0%)
	Untreated	394	41.9%	
Treated status in last 12 months	Treated	432	46%	2 (0.2%)
	Untreated	507	54%	
Treatment status for CLL at the time of COVID-19	Treated	320	34%	1 (0.1%)
	Untreated	620	66%	
Management of CLL treatment	Continued as planned	104	32.7%	(0.6%)
	Replaced with other	2	0.6%	
	Stopped	212	66.7%	
Total prior lines of treatment	1	275	50.7%	5 (0.9%)
	2	149	27.5%	
	3	60	11.1%	
	4	33	6.1%	
	>4	25	4.6%	
Treatment at the time of COVID-19	BTKi	179	56.3%	2 (0.6%)
	Venetoclax	34	10.7%	
	Venetoclax +Anti-CD20	17	5.3%	
	PI3K inhibitors	10	3.1%	
	PI3K inhibitors + Anti-CD20	3	0.9%	
	Anti-CD20	8	2.5%	
	Chemotherapy	22	6.9%	
	Chemoimmunotherapy	29	9.1%	
	BTKi + Venetoclax	7	2.2%	
	BTKi + Venetoclax + Anti-CD20	2	0.6%	
	Steroids only	7	2.2%	

*PI3K* Phosphatidylinositol-3 kinase, *BTKi* Bruton’s tyrosine kinase inhibitor.

### COVID-19 manifestations and management

The majority of patients (887/941, 94.3%) were symptomatic at the time of documented SARS-COV-2 infection. Fever was the most common COVID-19 related symptom (Supplemental Table 2).

In total, 237/941 (25.3%) patients were confined at home, while 695/941 (74.7%) patients were admitted to the hospital. Of the hospitalized patients, 177 (25.5%) were admitted to the ICU, 440 (63.3%) needed oxygen supplementation outside the ICU, and 78 (11.2%) were hospitalized without need of oxygen (Table 3).

**Table 3:** COVID-19 management, complications, and outcome.

			Number	Percentage	Missing
Measures taken for management of COVID-19		Intensive care	177	19%	9 (1%)
		Hospitalization with need of oxygen	440	47.2%	
		Hospitalization without need of oxygen	78	8.4%	
		Confinement at home only	237	25.4%	
Disease severity		Severe	617	66.2%	9 (1%)
		Nonsevere	315	33.8%	
Hospitalization		Hospitalization	695	74.6%	9 (1%)
		Home	237	25.4%	
Antiviral			266	35.7%	195 (20.7%)
Hydroxycloroquine or similar			203	27.1%	192 (20.4%)
Azithromycin			265	35.9%	202 (21.5%)
Steroids			514	61.3%	103 (10.9%)
Anti-IL6/IL6R			84	11.4%	204 (21.7%)
Convalescent/ Hyperimmune plasma			44	7.5%	351 (37.3%)
Pneumonia			659	75.4%	67 (7.1%)
Complications of COVID-19 infection		DIC	7	0.9%	167 (17.7%)
		VTE	48	6.2%	
PE (only for 48 with VTE)			40	87%	2 (4.2%)
Infection outcome	Resolution		647	69%	3 (0.3%)
	Still under medical care		34	3.6%	
	Death		257	27.4%	

*DIC* disseminated intravascular coagulation, *VTE* venous thromboembolism, *PE* pulmonary embolism.

Most hospitalized patients received pharmacologic treatment for COVID-19. Corticosteroids were the most commonly used treatment. In most cases (212/320, 66.2%), CLL-directed treatment was stopped during COVID-19 infection (Table 2). Signs of pneumonia were detected in 659/874 patients (75.4%). Forty-eight patients (6.2%) developed venous thromboembolism (40/48 pulmonary embolism) (Table 3).

### COVID-19 severity

Age, CIRS score, hypogammaglobulinemia, and specific comorbidities (COPD, coronary artery disease, diabetes, chronic renal disease) were identified as risk factors of severity in the univariate analyses. In the multivariate model, age (OR = 1.04; 95% CI: 1.02–1.06; *p* < 0.001), hypogammaglobulinemia (OR = 1.69; 95% CI: 1.20–2.38; *p* = 0.002), and coronary artery disease (OR = 2.83; 95% CI: 1.37–6.61; *p* = 0.009) remained statistically significant (Supplemental Table 3).

CLL treatment did not affect the severity of COVID-19. No difference was found in terms of severity between patients receiving BTKi at the time of COVID-19 versus untreated patients. Among 179 patients receiving BTKi at the time of COVID-19, age (OR = 1.04; 95% CI: 1.01–1.08; *p* = 0.017) and hypogammaglobulinemia (OR = 2.21; 95% CI: 1.13–4.36; *p* = 0.021) remained statistically significant predictors in the multivariate analysis (Supplemental Table 4).

### Overall survival and risk factors of fatality

In total, the CFR for all patients was 27.3% (257/941) (Supplemental Fig. 2). In 647 patients (69%) the infection resolved, 34 were under medical care at the time of data collection and in 3 the infection outcome was missing (Table 3). The vast majority of deaths occurred in patients with severe COVID-19 (236/617). Only 15 deaths occurred amongst 315 patients with nonsevere COVID-19 (CFR: 4.8%).

The baseline characteristics were similar between the two waves, except for age (*p* < 0.001) (Supplementary Tables 5, 6, and 7). The median age in the first wave was 71 years (with 39.9% older than 75 years) while in the second wave it was 68 years (with 27% older than 75 years). No difference in CFR was found between the first and second wave of COVID-19 in both all patients or only in patients with severe COVID-19, even after controlling for age *(p* = 0.78; *p* = 0.16, respectively) (Supplemental Tables 5 and 6).

In order to avoid collection bias, we restricted the analysis for both infection outcome and OS to patients with severe COVID-19 infection. We also excluded patients who were still under medical care and those missing information about the infection outcome.

The CFR for patients with severe COVID-19 was 38.4% (Supplemental Fig. 3). Through univariate analyses, age, unmutated IGHV gene somatic hypermutation status, specific comorbidities, and treatment status were observed as statistically significant risk factors for mortality. Age (OR = 1.04; 95% CI: 1.02–1.06; *p* < 0.001), cardiac failure (OR = 3.82; 95% CI: 1.31–13.94; *p* = 0.023) and treatment in the last 12 months (OR = 2.13; 95% CI: 1.44–3.20; *p* < 0.001) remained statistically significant in multivariate analysis (Table 4). We performed the same analysis including only the patients with severe COVID-19 that were reported from centers that participated in both studies. In the multivariate analysis, age and CLL treatment status remained statistically significant risk factors (Supplemental Table 8).

**Table 4:** Risk factors of infection outcome for patients with severe COVID-19 (*n* = 594).

Risk factor	Categories	Infection outcome		RR	*p* value
		Resolution	Death		
Age	≥ 65	240 (57.1%)	180 (42.9%)	1.33	0.02
	<65	118 (67.8%)	56 (32.2%)		
Age	≥75	104 (47.5%)	115 (52.5%)	1.63	<0.001
	<75	254 (67.7%)	121 (32.3%)		
Gender	Male	117 (60.9%)	75 (39.1%)	0.98	0.89
	Female	241 (60%)	161 (40%)		
IGHV gene somatic hypermutation status	Mutated^a^	91 (66.4%)	46 (33.6%)	0.70	0.017
	Unmutated^b^	91 (52.3%)	83 (47.7%)		
del(13q) (last assessment)	Negative	113(56.5%)	87 (43.5%)	1.14	0.37
	Positive	103 (61.7%)	64 (38.3%)		
del(11q) (last assessment)	Negative	185 (60.3%)	122 (39.7%)	0.83	0.3
	Positive	34 (52.3%)	31 (47.7%)		
trisomy 12 (last assessment)	Negative	176 (60.5%)	115 (39.5%)	0.87	0.45
	Positive	36 (54.5%)	30 (45.5%)		
del(17p) (last assessment)	Negative	206 (59.7%)	139 (40.3%)	0.75	0.09
	Positive	23 (46%)	27 (54%)		
*TP53* mutation status	Mutated	18 (48.6%)	19 (51.4%)	1.23	0.37
	Unmutated	137 (58.1%)	99 (41.9%)		
del(17p) positive and/or TP53 mutation	Yes	33 (50%)	33 (50%)	1.2	0.29
	No	130 (58.3%)	93 (41.7%)		
CIRS score	≤6	242 (65.4%)	128 (34.6%)	0.68	<0.001
	>6	87 (49.4%)	89 (50.6%)		
Other respiratory	Yes	24 (60%)	16 (40%)	1	>0.99
	No	332 (60.1%)	220 (39.9%)		
Asthma	Yes	10 (66.7%)	5 (33.3%)	0.83	0.8
	No	346 (60%)	231 (40%)		
COPD	Yes	22 (47.8%)	24 (52.2%)	1.35	0.11
	No	334 (61.2%)	212 (38.8%)		
Cardiac Failure	Yes	5 (25%)	15 (75%)	1.94	0.002
	No	351 (61.4%)	221 (38.6%)		
Arrythmias	Yes	33 (57.9%)	24 (42.1%)	1.06	0.83
	No	323(60.4%)	212(39.6)		
Coronary artery disease	Yes	32 (44.4%)	40 (55.6%)	1.47	0.006
	No	324 (62.3%)	196 (37.7%)		
Other cardiovascular	Yes	32 (54.2%)	27 (45.8%)	1.17	0.4
	No	324 (60.8%)	209 (39.2%)		
Hypertension	Yes	167 (56.8%)	127 (43.2%)	1.18	0.12
	No	189 (63.4%)	109 (36.6%)		
Diabetes	Yes	80 (64%)	45 (36%)	0.88	0.37
	No	276 (59.1%)	191 (40.9%)		
Chronic renal disease	Yes	15 (40.5%)	22 (59.5%)	1.54	0.019
	No	341 (61.4%)	214 (38.6%)		
Other hematological malignancies	Yes	5 (55.6%)	4 (44.4%)	1.16	0.75
	No	351 (60.2%)	232 (39.8%)		
Other non-hematological malignancies (excluding skin)	Yes	27 (61.4%)	17 (38.6%)	0.97	0.99
	No	329 (60%)	219 (40%)		
Obesity (BMI>30)	Yes	57 (61.3%)	36 (38.7%)	0.99	>0.99
	No	280 (61.1%)	178 (38.9%)		
Smoking	Current smoker	25 (59.5%)	17 (40.5%)		0.58
	Ex-smoker	82 (57.7%)	60 (42.3%)		
	Never	212 (62.7%)	126 (37.3%)		
Hypogammaglobulinemia (IgG <550 mg/dL)	Present	133 (55.6%)	106 (44.4%)	1.33	0.017
	Absent	146 (67%)	72 (33%)		
CLL treatment status	Treated	168 (49.9%)	169 (50.1%)	1.92	<0.001
	Untreated	190 (73.9%)	67 (26.1%)		
CLL treatment status at the time of COVID-19	Treated	94 (47.7%)	103 (52.3%)	1.56	<0.001
	Untreated	263 (66.4%)	133 (33.6%)		
Treated in last 12 months	Yes	132 (48.4%)	141 (51.6%)	1.42	0.002
	No	116 (63.7%)	66 (36.3%)		
Antiviral	Yes	140 (63.6%)	80 (36.4%)	1.07	0.65
	No	177 (66%)	91 (34%)		
Hydroxycloroquine or similar	Yes	116 (67.1%)	57 (32.9%)	0.92	0.61
	No	200 (64.3%)	111 (35.7%)		
Azithromycin	Yes	125 (69.4%)	55 (30.6%)	0.84	0.23
	No	187 (63.6%)	107 (36.4%)		
Steroids	Yes	264 (63%)	155 (37%)	1.04	0.88
	No	67 (64.4%)	37 (35.6%)		
Anti-IL6/IL6R	Yes	55 (66.3%)	28 (33.7%)	0.95	0.87
	NO	257(64.6%)	141 (35.4%)		
Country	Italy	108(61.7%)	67(38.3%)		0.09
	Spain	80(67.8%)	38(32.2%)		
	Other	170(56.5%)	131(43.5%)		

Multivariate analysis			
Risk factor	OR	95% CI	*p* value
Age (years)	1.04	1.02–1.06	<0.001
Cardiac failure (YES vs NO)	3.82	1.31–13.94	0.023
Treated in last 12 months (Treated vs Untreated)	2.13	1.44–3.20	<0.001

*IGHV* immunoglobulin heavy variable, *CIRS* cumulative illness rating scale, *COPD* chronic obstructive pulmonary disease, *RR* relative risk of death.

^a^Mutated: < 98% germline identity.

^b^Unmutated: ≥98% germline identity.

Univariate and multivariate analyses of risk factors of OS are outlined in Table 5. Older age (HR = 1.03; 95% CI: 1.02–1.04; *p* < 0.001), cardiac failure (HR = 1.79; 95% CI: 1.04–3.07; *p* = 0.035) and treatment status (HR = 0.54; 95% CI: 0.41–0.72; *p* < 0.001) appeared as statistically significant risk factors of OS in the multivariate analysis.

**Table 5:** Risk factors of OS.

**Risk factor**			***p*** **value**
Age (≥65 vs <65)			0.01
Age (≥75 vs <75)			<0.001
Gender (Male vs Female)			0.56
IGHV gene somatic hypermutation status (unmutated^a^ vs mutated^b^)			0.01
del(13q) (last assessment) (positive vs negative)			0.46
del(11q) (last assessment) (positive vs negative)			0.33
trisomy 12 (last assessment) (positive vs negative)			0.59
del(17p) (last assessment) (positive vs negative)			0.02
*TP53* mutation status (unmutated vs mutated)			0.18
del(17p) positive and/or *TP53* mutation (yes vs no)			0.1
CIRS score (> 6 vs ≤6)			<0.001
Other respiratory (YES vs NO)			0.82
Asthma (yes vs no)			0.49
COPD (yes vs no)			0.12
Cardiac Failure (yes vs no)			<0.001
Arrythmias (yes vs no)			0.38
Coronary artery disease (yes vs no)			0.06
Other cardiovascular (yes vs no)			0.16
Hypertension (yes vs no)			0.1
Diabetes (yes vs no)			0.52
Chronic renal disease (yes vs no)			0.02
Other hematological malignancies (yes vs no)			0.91
Other non-hematological malignancies (yes vs no)			0.87
Obesity (BMI>30) (yes vs no)			0.98
Smoking			0.8
Hypogammaglobulinemia (IgG <550 mg/dL) (present vs absent)			0.08
CLL treatment status (untreated vs treated)			<0.001
CLL treatment during COVID-19 (treated vs untreated)			<0.001
Treated in last 12 months (treated vs untreated)			0.01
Multivariate analysis			
**Risk factor**	**HR**	**95% CI**	
**Age (years)**	1.03	1.02–1.04	<0.001
Cardiac failure (yes vs no)	1.79	1.04–3.07	0.04
CLL treatment status (untreated vs treated)	0.54	0.41–0.72	<0.001

*IGHV* immunoglobulin heavy variable, *CIRS* cumulative illness rating scale, *COPD* chronic obstructive pulmonary disease.

^a^Unmutated: ≥98% germline identity.

^b^Mutated: <98% germline identity.

**Fig. 1:**
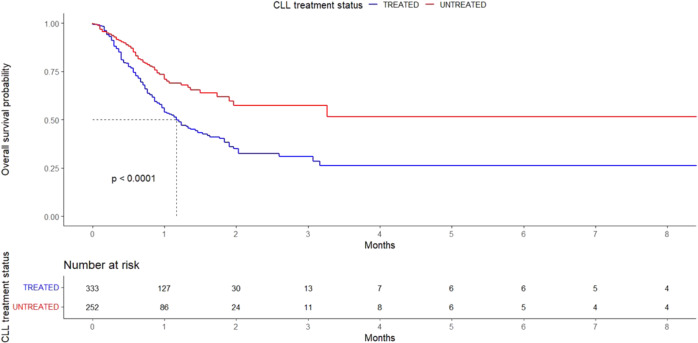
Overall survival in patients with severe COVID-19. Overall survival comparison between treated and untreated patients with CLL and severe COVID-19.

Comparison of OS between treated and untreated patients is depicted graphically in Fig. 1.

### CLL-directed treatment and COVID-19 outcome

**Fig. 2:**
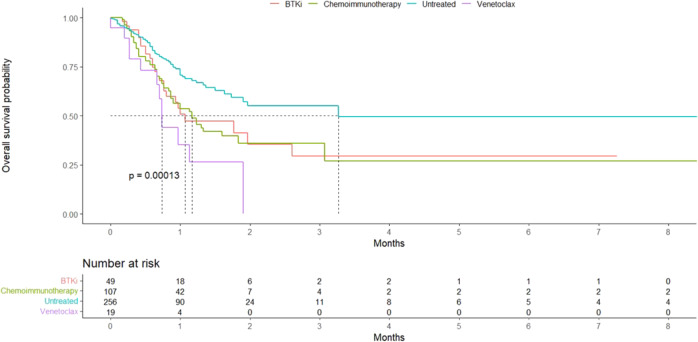
Overall survival in patients with severe COVID-19 according to treatment. Overall survival comparisons between patients treated with BTKi (at time of COVID-19), Venetoclax (at time of COVID-19), Chemoimmunotherapy in last 12 months, and Untreated.

We assessed the impact of specific treatment categories on COVID-19 outcome. Initially, we compared patients receiving BTKi at the time of SARS-COV-2 infection (*n* = 169) with patients receiving venetoclax at the same time (*n* = 31) and patients who had received chemoimmunotherapy in the last 12 months (*n* = 85). Then, in a second comparison, we kept the first two categories and replaced the third one with patients who had received any anti-CD20-based therapy (alone or in combination) in the last 12 months (*n* = 128). No statistically significant difference between the groups was noted in either analysis. Nonetheless, treated patients (in any treatment category) had a worse OS when compared to the untreated ones (*p* < 0.001) (Fig. 2 and Supplemental Fig. 4).

One hundred and ten patients with severe COVID-19 were being treated with BTKi monotherapy at the time of COVID-19. Thirty patients continued the BTKi treatment, while in 78 cases physicians chose to hold the drug. Patients who continued BTKi treatment did not have a statistically better outcome than those who discontinued the treatment (*p* = 0.08), while both groups had a worse outcome compared with untreated patients (*p* < 0.001) (Table 6).

**Table 6:** Outcome in patients with severe COVID-19.

Category	Infection outcome		*p* value
	Resolution	Death	
Untreated	190 (73.9%)	67 (26.1%)	<0.001
Continued BTKi	20 (66.7%)	10 (33.3%)	
Stopped BTKi	36 (46.2%)	42 (53.8%)	

No statistically significant difference between continued and stopped (*p* = 0.08).

*BTKi* Bruton’s tyrosine kinase inhibitor.

## Discussion

We here present the largest cohort published to date of patients with CLL and COVID-19. In this homogeneous disease-specific series, age, history of cardiac failure, and CLL treatment were the main risk factors for a dismal outcome.

In our study, the finding that older patients with CLL often have severe disease, higher CFRs, and lower survival time is in line with data derived from the general population, where COVID-19-related fatality and time to death positively correlated with age [3, 23, 24]. Relevant to add, similar results have been reported in a US study by Mato et al. showing that patients with CLL older than 75 years had a worse OS [15]. Older patients were also more likely to die in our first cohort, albeit without reaching statistical signifigance, which can be attributed to the smaller sample.

In our original cohort including patients from the first wave of SARS-CoV-2 [14], higher CIRS score and certain comorbidities were linked with increased mortality, whereas in this updated cohort only cardiac failure remained a statistically significant comorbidity in the multivariate analysis. OS was unaffected by diabetes, respiratory diseases, and obesity, variables conferring a worse prognosis in COVID-19 patients when analyzed in the general population [2, 3]. Nonetheless, some caution is warranted regarding the precise impact of specific comorbidities on COVID-19 outcome in patients with CLL since information on their severity was not collected systematically.

In this updated cohort, patients treated for CLL had a worse OS than untreated patients, while in both the report by Mato et al (198 patients, end point: outcome) and our previous report (190 patients, end point: severity), no difference was found between treated and untreated patients, probably due to the lower number of cases [14, 15]. Of note, when our previous cohort was combined with the Spanish cohort (281 patients), untreated patients had better OS than treated ones [16]. Thus, these apparent discrepancies may simply reflect the larger sample of our current analysis that allowed to identify a difference between the untreated and the treated, likely more immunosuppressed, patients.

Untreated patients had a better outcome also when compared separately with patients in different treatment categories (BTKi, venetoclax, chemoimmunotherapy, or anti-CD20 containing regimens). Patients who continued BTKi treatment during COVID-19 infection did not appear to fare worse than those who stopped the drug with a potential benefit albeit not statistically significant, suggesting that BTKi continuation definitely is not harming patients and may potentially benefit them by preventing respiratory failure and death or by keeping CLL under control [25]. This is in line with the current recommendations indicating no need to hold BTKi at the time of a confirmed SARS-CoV-2 infection [26].

Patients treated with anti-CD20 antibodies, alone or in combination, had worse OS than untreated patients. This finding suggests that such treatment likely renders patients with CLL more susceptible to succumb to COVID-19 infection. That said, the small number of patients treated with the combination of anti-CD20 antibodies with novel agents in the present cohort hinders firm conclusions from being drawn regarding its precise impact on COVID-19 outcome.

The CFR in the entire cohort was 27.4% and increased to 38.4% among patients with severe COVID-19. This indicates a remarkable consistency among different studies in patients with CLL and COVID-19 as well as in the different waves of the pandemic, confirming the unabated aggressiveness of the disease with time [14–16]. In the general population, the CFR in hospitalized patients appears lower compared to our cohort: indicatively, a meta-analysis of COVID-19 patients found a fatality rate of 17.1% and 40.5% for hospitalized and critically ill patients, respectively [27]. Similarly, in-hospital mortality of patients older than 75 years in a US study was 20.5% [28]. Nevertheless, the caveats of cross-study comparisons, the retrospective nature of our cohort, and the lack of a control group hinder any generalization about the true impact of CLL in COVID-19-related mortality.

In the updated cohort, CLL-directed treatment was associated with a worse outcome. However, it had no impact on COVID-19 severity in either the orginal or the updated cohort. We suspect that investigators were more likely to be informed about patients on CLL-specific treatment with mild COVID-19 symptoms than untreated patients, since the former were followed up more closely and probably sought immediate guidance: thus, we may have missed more asymptomatic untreated patients.

Dexamethasone and tocilizumab remain the only treatments that showed a mortality benefit in patients with COVID-19 [29, 30]. The impact of COVID-19 treatments in our cohort was not among the objectives, with information on the administered treatment for COVID-19 missing at least in part. Nonetheless, we observed that different types of therapies against COVID-19 did not affect the CFR.

We acknowledge certain limitations in our study. As already mentioned, hospitalized and/or symptomatic patients were more likely to be captured in our cohort, while we probably missed some asymptomatic or mildly symptomatic patients who avoided undergoing testing and/or contacting their physician, especially during the first wave, when COVID-19 testing was not the rule. To avoid the caveats of a retrospective study and the lack of a control group in overestimating the mortality, we restricted the analysis to patients with severe disease and avoided broad generalizations about prevalence, morbidity, and mortality. Taking into account the multicenter, international design of the study, some heterogeneity in our patient population is to be expected as we included patients from different countries, diagnosed with COVID-19 during different waves of the pandemic and managed differently. Along this line, mirroring the course of the pandemic, some countries like Italy and Spain were overrepresented in the first wave, while the second wave featured more patients from other countries (e.g., Czech Republic and Greece). Finally, we recognize that differences in the quality of health care and weather could influence our results. Indeed, only a small number of cases were diagnosed during the summer and none of the patients in our cohort was vaccinated for SARS-CoV-2. In addition, the lack of any difference in CFRs between the two waves illustrates that CLL and treatment-related immunosuppression may overshadow other risk factors of outcome.

Taken together, our findings suggest that in patients with CLL and COVID-19, older age confers a worse prognosis, with increased mortality. Untreated patients had a better chance of survival than those on treatment or recently treated.

## Supplementary information

Supplemental information

## Data Availability

All data created during this study are included in the manuscript and supporting material. The code for biostatistical analysis is available in the following GitHub repository: https://github.com/gkarakatsoulis/COVID-19-in-CLL-patients.
