# Supplementary material for: COVID-19 severity and mortality in patients with CLL: an update of the international ERIC and Campus CLL study
Source: Leukemia. 2021 Nov 1;35(12):3444–54. doi: 10.1038/s41375-021-01450-8 (PMC8559135; doi:10.1038/s41375-021-01450-8)

**Supplemental information**

**Table 1. Country of origin and number of participating sites.**

| **Country** | **Number of sites providing clinical data (n=91)** | **Number of cases**  **(n=1009)** |
| --- | --- | --- |
| Italy (CLL Campus) | 27 | 296 |
| Spain | 13 | 161 |
| The Netherlands | 7 | 43 |
| Israel | 5 | 19 |
| Greece | 4 | 27 |
| United Kingdom (Great Britain/ Northern Ireland) | 4 (3/1) | 25 |
| Czech Republic | 3 | 103 |
| Poland | 3 | 55 |
| Denmark | 3 | 22 |
| Russian Federation | 2 | 52 |
| Romania | 2 | 42 |
| Serbia | 2 | 22 |
| Argentina | 2 | 16 |
| Germany | 2 | 12 |
| Belgium | 2 | 8 |
| Croatia | 1 | 31 |
| Sweden | 1 | 20 |
| Portugal | 1 | 12 |
| Armenia | 1 | 11 |
| Switzerland | 1 | 11 |
| Egypt | 1 | 8 |
| India | 1 | 5 |
| Qatar | 1 | 5 |
| Ireland | 1 | 2 |
| Nepal | 1 | 1 |

**Table 2. COVID-19 Symptoms and laboratory examinations.**

| **Symptoms** | **Frequency** | **Percentage** | **Missing** |
| --- | --- | --- | --- |
| Fever | 677 | 75.1% | 40 (4.3%) |
| Dyspnea | 391 | 43.6% | 44 (4.7%) |
| Cough | 463 | 51.3% | 38 (4%) |
| Fatigue | 214 | 23.7% | 38 (4%) |
| Headache | 55 | 6.1% | 37 (3.9%) |
| GI symptoms | 88 | 9.7% | 37 (3.9%) |
| Anosmia/ Ageusia | 66 | 7.3% | 37 (3.9%) |
| Myalgias/ Arthalgias | 77 | 8.5% | 37 (3.9%) |
| Other | 155 | 17.2% | 38 (4%) |

| **Laboratory value** | **Median** | **Min** | **Max** | **Missing** |
| --- | --- | --- | --- | --- |
| CRP x times above uln | 20 | 0 | 160 | 418 (44.4%) |
| D-DIMERS x times above uln | 2.8 | 0.162 | 100 | 502 (53.3%) |
| ALC (x10^9/l) | 12.4 | 0.04 | 675.43 | 321 (34.1%) |

GI: Gastrointestinal, CRP: C-reactive protein, ULN: Upper limit of normal, ALC: Absolute lymphocyte number

**Table 3. Disease severity in all patients.**

| **Risk factor** | Categories | | Disease | | |  |
| --- | --- | --- | --- | --- | --- | --- |
|  |  |  | Non-severe | | Severe | p-value |
| Age | $\geq65$ | | 163 (27.3%) | | 434 (72.7%) | **<0.001** |
|  | $<65$ | | 152 (45.4%) | | 183 (54.6%) |  |
| Age | $\geq75$ | | 64 (22%) | | 227 (78%) | **<0.001** |
|  | $<75$ | | 251 (39.2%) | | 390 (60.8%) |  |
| Gender | Male | | 202 (32.4%) | | 421 (67.6%) | 0.24 |
|  | Female | | 113 (36.6%) | | 196 (63.4%) |  |
| IGHV gene somatic hypermutation status | Mutated* | | 88 (38.3%) | | 142 (61.7%) | >0.99 |
|  | Unmutated** | | 114 (38.1%) | | 185 (61.9%) |  |
| del(13q) | Negative | | 113 (35.2%) | | 208 (64.8%) | >0.99 |
|  | Positive | | 95 (35.1%) | | 176 (64.9%) |  |
| del(11q) | Negative | | 179 (35.9%) | | 319 (64.1%) | 0.783 |
|  | Positive | | 36 (34%) | | 70 (66%) |  |
| trisomy 12 | Negative | | 174 (36.3%) | | 305(63.7%) | 0.17 |
|  | Positive | | 27 (28.4%) | | 68 (71.6%) |  |
| del(17p) | Negative | | 197 (35.4%) | | 359 (64.6%) | 0.78 |
|  | Positive | | 32 (37.6%) | | 53 (62.4%) |  |
| *TP53* mutation status | Mutated | | 28 (41.8%) | | 39 (58.2%) | 0.26 |
|  | Unmutated | | 127(33.8%) | | 250 (66.2%) |  |
| del17p positive or  TP53 mutation | YES | | 47 (40.5%) | | 69 (59.5%) | 0.22 |
|  | NO | | 119 (33.7%) | | 234 (66.3%) |  |
| CIRS score | $\leq6$ | | 222 (36.4%) | | 388 (63.6%) | **0.012** |
|  | $>6$ | | 67 (27.1%) | | 180 (72.9%) |  |
| Other respiratory | YES | | 16 (26.2%) | | 45 (73.8%) | 0.25 |
|  | NO | | 297 (34.3%) | | 570 (65.7%) |  |
| Asthma | YES | | 6 (28.6%) | | 15 (71.4%) | 0.79 |
|  | NO | | 307 (33.8%) | | 600 (66.2%) |  |
| COPD | YES | | 11 (19%) | | 47 (81%) | **0.02** |
|  | NO | | 302 (34.7%) | | 568 (65.3%) |  |
| Cardiac Failure | YES | | 8 (27.6%) | | 21 (72.4%) | 0.61 |
|  | NO | | 305 (33.9%) | | 594 (66.1%) |  |
| Arrythmias | YES | | 25 (29.1%) | | 61 (70.9%) | 0.40 |
|  | NO | | 288 (34.2%) | | 554 (65.8%) |  |
| Coronary artery disease | YES | | 14 (16.1%) | | 73 (83.9%) | **<0.001** |
|  | NO | | 299 (35.6%) | | 542 (64.4%) |  |
| Other cardiovascular | YES | | 23 (28%) | | 59 (72%) | 0.31 |
|  | NO | | 290 (34.3%) | | 556 (65.7%) |  |
| Hypertension | YES | | 132 (30.4%) | | 302 (69.6%) | 0.053 |
|  | NO | | 181 (36.6%) | | 313 (63.4%) |  |
| Diabetes | YES | | 44 (25.6%) | | 128 (74.4%) | **0.02** |
|  | NO | | 269 (35.6%) | | 487 (64.4%) |  |
| Chronic renal disease | YES | | 9 (18.4%) | | 40 (81.6%) | **0.03** |
|  | NO | | 304 (34.6%) | | 575 (65.4%) |  |
| Other hematological malignancies | YES | | 1 (10%) | | 9 (90%) | 0.18 |
|  | NO | | 312 (34%) | | 606 (66%) |  |
| Other non-hematological malignancies (excluding skin) | YES | | 27 (36%) | | 48 (64%) | 0.76 |
|  | NO | | 286 (33.5%) | | 567(66.5%) |  |
| Obesity (BMI>30) | YES | | 54 (35.8%) | | 97 (64.2%) | 0.59 |
|  | NO | | 235 (33.1%) | | 476 (66.9%) |  |
| Smoking | Current smoker | | 24 (34.8%) | | 45 (65.2%) | 0.9 |
|  | Ex-smoker | | 78 (35.1%) | | 144 (64.9%) |  |
|  | Never | | 177 (33.5%) | | 352 (66.5%) |  |
| Hypogammaglobulinemia  (IgG <550 mg/dL) | Present | | 99 (28.1%) | | 253 (71.9%) | **0.01** |
|  | Absent | | 136 (37.9%) | | 223 (62.1%) |  |
| CLL treatment status | Treated | | 190 (34.9%) | | 355 (65.1%) | 0.46 |
|  | Untreated | | 125 (32.3%) | | 262 (67.7%) |  |
| CLL treatment status at the time of COVID-19 | Treated | | 113 (35.3%) | | 207 (64.7%) | 0.537 |
|  | Untreated | | 202 (33.1%) | | 409 (66.9%) |  |
| Treated in last 12 months (with the untreated) | YES | | 144 (33.4%) | | 287 (66.6%) | 0.89 |
|  | NO | | 170 (34.1%) | | 329 (65.9%) |  |
| Treated in last 12 months (only for treated) | YES | | 144 (33.4%) | | 287 (66.6%) | 0.22 |
|  | NO | | 45 (40.2%) | | 67 (59.8%) |  |
| **Multivariate analysis** | | | | | | |
| **Risk factor** | | **OR** | | **95% CI** | | **p-value** |
| Age (years) | | 1.04 | | 1.02 – 1.06 | | **<0.001** |
| Coronary artery disease (YES vs NO) | | 2.83 | | 1.37 – 6.61 | | **0.01** |
| Hypogammaglobulinemia  (IgG <550 mg/dL) (Present vs Absent) | | 1.69 | | 1.20 – 2.38 | | **0.002** |

*Mutated: <98% germline identity, **Unmutated: ≥98% germline identity

IGHV: immunoglobulin heavy variable, CIRS: cumulative illness rating scale, COPD: chronic obstructive pulmonary disease

**Table 4. Severity only for BTKi patients.**

| **Risk factor** | **Categories** | **Severity** | | | **p-value** |
| --- | --- | --- | --- | --- | --- |
|  |  | **Serious** | | **Non Serious** |  |
| Age | $\geq65$ | 89 (66.9%) | | 44 (33.1%) | **0.01** |
|  | $<65$ | 25 (45.5%) | | 30 (54.5%) |  |
| Age | $\geq75$ | 41 (71.9%) | | 16 (28.1%) | 0.054 |
|  | $<75$ | 73 (55.7%) | | 58 (44.3%) |  |
| Gender | Male | 84 (62.7%) | | 50 (37.3%) | 0.459 |
|  | Female | 30 (55.6%) | | 24 (44.4%) |  |
| IGHV gene somatic hypermutation status | Mutated* | 23 (57.5%) | | 17 (42.5%) | 0.99 |
|  | Unmutated** | 59 (55.7%) | | 47 (44.3%) |  |
| del(13q) (last assessment) | Negative | 51 (60%) | | 34 (40%) | >0.99 |
|  | Positive | 47 (60.3%) | | 31 (39.7%) |  |
| del(11q) (last assessment) | Negative | 72 (60%) | | 48 (40%) | >0.99 |
|  | Positive | 27 (58.7%) | | 19 (41.3%) |  |
| trisomy 12 | Negative | 77 (57.9%) | | 56 (42.1%) | 0.18 |
|  | Positive | 20 (74.1%) | | 7 (25.9%) |  |
| del(17p) (last assessment) | Negative | 78 (58.6%) | | 55 (41.4%) | 0.89 |
|  | Positive | 21 (61.8%) | | 13 (38.2%) |  |
| *TP53* mutation status | Mutated | 13 (46.4%) | | 15 (53.6%) | 0.28 |
|  | Unmutated | 66 (60%) | | 44 (40%) |  |
| del17p positive and/or  *TP53* mutation | YES | 24 (53.3%) | | 21 (46.7%) | 0.55 |
|  | NO | 59 (60.2%) | | 39 (39.8%) |  |
| CIRS score | $\leq6$ | 64 (57.7%) | | 47 (42.3%) | 0.085 |
|  | $>6$ | 38 (73.1%) | | 14 (26.9%) |  |
| Other respiratory | YES | 12 (75%) | | 4 (25%) | 0.34 |
|  | NO | 101(59.4%) | | 69 (40.6%) |  |
| Asthma | YES | 2 (50%) | | 2 (50%) | 0.65 |
|  | NO | 111 (61%) | | 71 (39%) |  |
| COPD | YES | 14 (77.8%) | | 4 (22.2%) | 0.19 |
|  | NO | 99 (58.9%) | | 69 (41.1%) |  |
| Cardiac Failure | YES | 1 (50%) | | 1 (50%) | >0.99 |
|  | NO | 112(60.9%) | | 72 (39.1%) |  |
| Arrythmias | YES | 9 (69.2%) | | 4 (30.8%) | 0.72 |
|  | NO | 104(60.1%) | | 69 (39.9%) |  |
| Coronary artery disease | YES | 16 (88.9%) | | 2 (11.1%) | **0.02** |
|  | NO | 97 (57.7%) | | 71 (42.3%) |  |
| Other cardiovascular | YES | 14 (77.8%) | | 4 (22.2%) | 0.19 |
|  | NO | 99 (58.9%) | | 69 (41.1%) |  |
| Hypertension | YES | 64 (69.1%) | | 30 (31.9%) | 0.055 |
|  | NO | 49 (53.3%) | | 43 (46.7%) |  |
| Diabetes | YES | 25 (73.5%) | | 9 (26.5%) | 0.14 |
|  | NO | 88 (57.9%) | | 64 (42.1%) |  |
| Chronic renal disease | YES | 11 (73.3%) | | 4 (26.7%) | 0.44 |
|  | NO | 102(59.6%) | | 69 (40.4%) |  |
| Other hematological malignancies | YES | 2 (100%) | | 0 (0%) | - |
|  | NO | 111(60.3%) | | 73 (39.7%) |  |
| Other non-hematological malignancies (excluding skin) | YES | 7 (43.8%) | | 9 (56.2%) | 0.23 |
|  | NO | 106(62.4%) | | 64 (37.6%) |  |
| Obesity (BMI>30) | YES | 22 (62.9%) | | 13 (37.1%) | >0.99 |
|  | NO | 88 (61.1%) | | 56 (38.9%) |  |
| Smoking | Current smoker | 8 (88.9%) | | 1 (11.1%) | 0.18 |
|  | Ex-smoker | 31 (56.4%) | | 24 (43.6%) |  |
|  | Never | 64 (59.8%) | | 43 (40.2%) |  |
| Hypogammaglobulinemia  (IgG <550 mg/dL) | Present | 64 (69.6%) | | 28 (30.4%) | **0.02** |
|  | Absent | 32 (49.2%) | | 33 (50.8%) |  |
| Antiviral | YES | 39 (75%) | | 13 (25%) | **0.01** |
|  | NO | 55 (51.4%) | | 52 (48.6%) |  |
| Hydroxycloroquine | YES | 33 (80.5%) | | 8 (19.5%) | **0.003** |
|  | NO | 62 (52.1%) | | 57 (47.9%) |  |
| Azithromycin | YES | 36 (66.7%) | | 18 (33.3%) | 0.25 |
|  | NO | 58 (55.8%) | | 46 (44.2%) |  |
| Steroids | YES | 80 (80.8%) | | 19 (19.2%) | **<0.001** |
|  | NO | 16 (23.5%) | | 52 (76.5%) |  |
| Anti-IL6/IL6R | YES | 20 (95.2%) | | 1 (4.8%) | **0.001** |
|  | NO | 76 (55.1%) | | 62 (44.9%) |  |
| **Multivariate analysis** | | | | | |
| **Risk factor** | | | **OR** | **95% CI** | **p-value** |
| Age (years) | | | 1.04 | 1.01 – 1.08 | **0.017** |
| Hypogammaglobulinemia (Present vs Absent) | | | 2.21 | 1.13 – 4.36 | **0.021** |

*Mutated: <98% germline identity, **Unmutated: ≥98% germline identity

IGHV: immunoglobulin heavy variable, CIRS: cumulative illness rating scale, COPD: chronic obstructive pulmonary disease, IL6: Interleukin 6, IL6R: Interleukin 6 Receptor

**Table 5. Comparisons of patients’ characteristics with severe COVID-19 between the two waves (n=593)**

Age:

First wave: median = 73 (64 – 80)

Second wave: median = 69 (63 – 79)

Number of comorbidities:

First: median = 2 (1 – 3)

Second: median = 2 (1 – 4)

| **Characteristic** | Categories | Wave | | p-value |
| --- | --- | --- | --- | --- |
|  |  | 1 | 2 |  |
| Age | $\geq65$ | 164(72.9%) | 255(69.3%) | 0.4 |
|  | $<65$ | 61(27.1%) | 113(30.7%) |  |
| Age | $\geq75$ | 97(43.1%) | 121(32.9%) | 0.016 |
|  | $<75$ | 128(56.9%) | 247(67.1%) |  |
| Gender | Male | 152(67.6%) | 249(67.7%) | >0.99 |
|  | Female | 73(32.4%) | 119(32.3%) |  |
| IGHV gene status | Mutated | 57(48.7%) | 80(41.2%) | 0.24 |
|  | Unmutated | 60(51.3%) | 114(58.8%) |  |
| del13q (last assessment) | Negative | 72(50.3%) | 128(57.4%) | 0.22 |
|  | Positive | 71(49.7%) | 95(42.6%) |  |
| del11q (last assessment) | Negative | 127(88.8%) | 179(78.5%) | 0.016 |
|  | Positive | 16(11.2%) | 49(21.5%) |  |
| trisomy 12 (last assessment) | Negative | 114(81.4%) | 176(81.5%) | >0.99 |
|  | Positive | 26(18.6%) | 40(18.5%) |  |
| del17p (last assessment) | Negative | 128(87.1%) | 216(87.4%) | >0.99 |
|  | Positive | 19(12.9%) | 31(12.6%) |  |
| TP53 mutation status | Mutated | 13(13%) | 24(13.9%) | 0.98 |
|  | Unmutated | 87(87%) | 149(86.1%) |  |
| del17p positive or  TP53 mutation | YES | 24(22.4%) | 42(23.1%) | >0.99 |
|  | NO | 83(77.6%) | 140(76.9%) |  |
| CIRS score | $\leq6$ | 149(72.7%) | 221(65%) | 0.08 |
|  | $>6$ | 56(27.3%) | 119(35%) |  |
| Other respiratory | YES | 22(9.8%) | 18(4.9%) | 0.03 |
|  | NO | 202(90.2%) | 349(95.1%) |  |
| Asthma | YES | 9(4%) | 6(1.6%) | 0.13 |
|  | NO | 215(96%) | 361(98.4%) |  |
| COPD | YES | 16(7.1%) | 30(8.2%) | 0.77 |
|  | NO | 208(92.1%) | 337(91.8%) |  |
| Cardiac Failure | YES | 11(4.9%) | 9(2.5%) | 0.17 |
|  | NO | 213(95.1%) | 358(97.5%) |  |
| Arrythmias | YES | 13(5.8%) | 44(12%) | 0.02 |
|  | NO | 211(94.2%) | 323(88%) |  |
| Coronary artery disease | YES | 24(10.7%) | 48(13.1%) | 0.47 |
|  | NO | 200(89.3%) | 319(86.9%) |  |
| Other cardiovascular | YES | 11(4.9%) | 48(13.9%) | 0.002 |
|  | NO | 213(95.1%) | 319(86.9%) |  |
| Hypertension | YES | 98(43.8%) | 195(53.1%) | 0.03 |
|  | NO | 126(56.2%) | 172(46.9%) |  |
| Diabetes | YES | 50(22.3%) | 75(20.4%) | 0.66 |
|  | NO | 174(77.7%) | 292(79.6%) |  |
| Chronic renal disease | YES | 10(4.5%) | 27(7.4%) | 0.22 |
|  | NO | 214(95.5%) | 340(92.6%) |  |
| Other hematological malignancies | YES | 1(0.4%) | 8(2.2%) | 0.16 |
|  | NO | 223(99.6%) | 359(97.8%) |  |
| Other non-hematological malignancies (excluding skin) | YES | 13(5.8%) | 31(8.4%) | 0.3 |
|  | NO | 211(94.2%) | 336(91.6%) |  |
| Obesity (BMI>30) | YES | 31(15.2%) | 67(17.1%) | 0.5 |
|  | NO | 173(84.8%) | 285(82.1%) |  |
| Smoking | Current smoker | 14(7.6%) | 28(8.3%) | 0.84 |
|  | Ex-smoker | 53(28.6%) | 89(26.4%) |  |
|  | Never | 118(63.8%) | 220(65.3%) |  |
| Hypogammaglobulinemia  (IgG <550 mg/dL) | Present | 86(48%) | 153(55.2%) | 0.16 |
|  | Absent | 93(52%) | 124(44.8%) |  |
| CLL treatment status | Treated | 120(53.3%) | 216(58.7%) | 0.23 |
|  | Untreated | 105(46.7%) | 152(41.3%) |  |
| CLL treatment status at the time of COVID-19 | Treated | 63(28%) | 134(36.5%) | 0.04 |
|  | Untreated | 162(72%) | 233(63.5%) |  |
| Treated in last 12 months (with the untreated) | YES | 94(55.3%) | 179(63%) | 0.13 |
|  | NO | 76(44.7%) | 105(37%) |  |
| Treated in last 12 months (only for treated) | YES | 94(78.3%) | 179(83.3%) | 0.33 |
|  | NO | 26(21.7%) | 36(16.7%) |  |
| Antiviral | YES | 111(53.1%) | 109(39.1%) | 0.003 |
|  | NO | 98(46.9%) | 170(60.9%) |  |
| Hydroxycloroquine or similar | YES | 165(79.3%) | 8(2.9%) | <0.001 |
|  | NO | 43(20.7%) | 268(97.1%) |  |
| Azithromycin | YES | 97(48.5%) | 83(30.3%) | <0.001 |
|  | NO | 103(51.5%) | 191(69.7%) |  |
| Steroids | YES | 128(64%) | 291(90.1%) | <0.001 |
|  | NO | 72(36%) | 32(9.9%) |  |
| Anti-IL6/IL6R | YES | 57(27.8%) | 26(9.4%) | <0.001 |
|  | NO | 148(72.2%) | 250(90.6%) |  |
| Infection outcome | Resolution | 142(63.1%) | 216(58.7%) | 0.33 |
|  | Death | 83(36.9%) | 152(41.3%) |  |

*Mutated: <98% germline identity, **Unmutated: ≥98% germline identity

IGHV: immunoglobulin heavy variable, CIRS: cumulative illness rating scale, COPD: chronic obstructive pulmonary disease, IL6: Interleukin 6, IL6R: Interleukin 6 Receptor

**Table 6. Comparison between the two waves, after controlling for age.**

| Category | OR | 95% CI | p-value |
| --- | --- | --- | --- |
| **All patients** | | | |
| Age (years) | 0.95 | 0.94 – 0.96 | <0.001 |
| Wave | 0.96 | 0.69 – 1.31 | 0.78 |
| **Only for severe patients** | | | |
| Age (years) | 0.97 | 0.95 – 0.98 | <0.001 |
| Wave | 0.78 | 0.55 – 1.10 | 0.16 |

**Table 7. Comparisons of patients’ characteristics with severe COVID-19 between the two waves only for the clinical sites participated in both periods of the study (n=314).**

Median age:

1^st^ wave: 73 (64 – 80.5)

2^nd^ wave: 71 (64 – 79)

Number of comorbidities:

1^st^ wave: 2 (1 – 3)

2^nd^ wave: 2 (1 – 4)

| **Characteristic** | Categories | Wave | | p-value |
| --- | --- | --- | --- | --- |
|  |  | 1 | 2 |  |
| Age | $\geq65$ | 111(73.5%) | 118(72.4%) | 0.92 |
|  | $<65$ | 40(26.5%) | 45(27.6%) |  |
| Age | $\geq75$ | 87(57.6%) | 104(63.8%) | 0.31 |
|  | $<75$ | 64(42.4%) | 59(36.2%) |  |
| Gender | Male | 103(68.2%) | 113(69.3%) | 0.93 |
|  | Female | 48(31.8%) | 50(30.7%) |  |
| IGHV gene status | Mutated | 44(47.8%) | 44(43.1%) | 0.61 |
|  | Unmutated | 48(52.2%) | 58(56.9%) |  |
| del13q (last assessment) | Negative | 55(50.5%) | 64(58.7%) | 0.28 |
|  | Positive | 54(49.5%) | 45(41.3%) |  |
| del11q (last assessment) | Negative | 96(88.1%) | 85(77.3%) | 0.053 |
|  | Positive | 13(11.9%) | 25(22.7%) |  |
| trisomy 12 (last assessment) | Negative | 93(85.3%) | 92(84.4%) | >0.99 |
|  | Positive | 16(14.7%) | 17(15.6%) |  |
| del17p (last assessment) | Negative | 94(84.7%) | 112(96.6%) | 0.004 |
|  | Positive | 17(15.3%) | 4(3.4%) |  |
| TP53 mutation status | Mutated | 9(12%) | 8(8.8%) | 0.67 |
|  | Unmutated | 66(88%) | 83(91.2%) |  |
| del17p positive or  TP53 mutation | YES | 19(23.2%) | 9(10.2%) | 0.04 |
|  | NO | 63(76.8%) | 79(89.8%) |  |
| CIRS score | $\leq6$ | 107(73.3%) | 95(65.1%) | 0.16 |
|  | $>6$ | 39(26.7%) | 51(34.9%) |  |
| Other respiratory | YES | 13(8.7%) | 10(6.1%) | 0.52 |
|  | NO | 137(91.3%) | 153(93.9%) |  |
| Asthma | YES | 5(3.3%) | 2(1.2%) | 0.27 |
|  | NO | 145(96.7%) | 161(98.8%) |  |
| COPD | YES | 9(6%) | 13(8%) | 0.64 |
|  | NO | 141(94%) | 150(92%) |  |
| Cardiac Failure | YES | 5(3.3%) | 3(1.8%) | 0.49 |
|  | NO | 145(96.7%) | 160(98.2%) |  |
| Arrythmias | YES | 8(5.3%) | 16(9.8%) | 0.2 |
|  | NO | 142(94.7%) | 147(90.2%) |  |
| Coronary artery disease | YES | 15(10%) | 11(6.7%) | 0.4 |
|  | NO | 135(90%) | 152(93.3%) |  |
| Other cardiovascular | YES | 9(6%) | 26(16%) | 0.009 |
|  | NO | 141(94%) | 137(84%) |  |
| Hypertension | YES | 66(44%) | 94(57.7%) | 0.02 |
|  | NO | 84(56%) | 69(42.3%) |  |
| Diabetes | YES | 30(20%) | 35(21.5%) | 0.86 |
|  | NO | 120(80%) | 128(78.5%) |  |
| Chronic renal disease | YES | 8(5.3%) | 10(6.1%) | 0.95 |
|  | NO | 142(94.7%) | 153(93.9%) |  |
| Other hematological malignancies | YES | 1(0.7%) | 3(1.8%) | 0.62 |
|  | NO | 149(99.3%) | 160(98.2%) |  |
| Other non-hematological malignancies (excluding skin) | YES | 10(6.7%) | 21(12.9%) | 0.1 |
|  | NO | 140(93.3%) | 142(87.1%) |  |
| Obesity (BMI>30) | YES | 24(16.4%) | 25(15.7%) | 0.99 |
|  | NO | 122(83.6%) | 134(84.3%) |  |
| Smoking | Current smoker | 5(3.9%) | 8(5.3%) | 0.71 |
|  | Ex-smoker | 37(28.7%) | 48(31.6%) |  |
|  | Never | 87(67.4%) | 96(63.1%) |  |
| Hypogammaglobulinemia  (IgG <550 mg/dL) | Present | 60(50%) | 62(48.8%) | 0.95 |
|  | Absent | 60(50%) | 65(51.2%) |  |
| CLL treatment status | Treated | 83(55%) | 84(51.5%) | 0.62 |
|  | Untreated | 68(45%) | 79(48.5%) |  |
| CLL treatment status at the time of COVID-19 | Treated | 42(27.8%) | 53(32.5%) | 0.43 |
|  | Untreated | 109(72.2%) | 110(67.5%) |  |
| Treated in last 12 months (with the untreated) | YES | 67(57.8%) | 73(62.9%) | 0.5 |
|  | NO | 49(42.2%) | 43(37.1%) |  |
| Treated in last 12 months (only for treated) | YES | 67(80.7%) | 73(86.9%) | 0.38 |
|  | NO | 16(19.3%) | 11(13.1%) |  |
| Antiviral | YES | 86(59.7%) | 52(36.4%) | <0.001 |
|  | NO | 58(40.3%) | 91(63.6%) |  |
| Hydroxycloroquine or similar | YES | 124(86.7%) | 2(1.4%) | <0.001 |
|  | NO | 19(11.3%) | 140(98.6%) |  |
| Azithromycin | YES | 80(57.6%) | 45(31.9%) | <0.001 |
|  | NO | 59(42.4%) | 96(68.1%) |  |
| Steroids | YES | 102(71.3%) | 136(90.1%) | <0.001 |
|  | NO | 41(28.7%) | 15(9.9%) |  |
| Anti-IL6/IL6R | YES | 45(31.5%) | 16(10.7%) | <0.001 |
|  | NO | 98(68.5%) | 133(89.3%) |  |
| Infection outcome | Resolution | 95(62.9%) | 108(66.3%) | 0.62 |
|  | Death | 56(37.1%) | 55(33.7%) |  |

*Mutated: <98% germline identity, **Unmutated: ≥98% germline identity

IGHV: immunoglobulin heavy variable, CIRS: cumulative illness rating scale, COPD: chronic obstructive pulmonary disease, IL6: Interleukin 6, IL6R: Interleukin 6 Receptor

**Table 8.** **Risk factors of infection outcome for patients with severe COVID-19, only for centers that participated in both studies (n= 315).**

| **Risk factor** | **Categories** | **Infection Outcome** | | | | | **RR** | **p-value** |
| --- | --- | --- | --- | --- | --- | --- | --- | --- |
|  |  | **Resolution** | | | **Death** | |  |  |
| Age | $\geq65$ | 142(61.7%) | | | 88(38.3%) | | 0.74 | 0.13 |
|  | $<65$ | 61(71.8%) | | | 24(28.2%) | |  |  |
| Age | $\geq75$ | 62(50%) | | | 62(50%) | | **0.52** | **<0.001** |
|  | $<75$ | 141(73.8%) | | | 50(26.2%) | |  |  |
| Gender | Male | 133(61.3%) | | | 84(38.7%) | | 0.74 | 0.11 |
|  | Female | 70(71.4%) | | | 28(28.6%) | |  |  |
| IGHV gene somatic hypermutation status | Mutated* | 57(64.8%) | | | 31(35.2%) | | 1.18 | 0.46 |
|  | Unmutated** | 62(58.5%) | | | 44(41.5%) | |  |  |
| del(13q) (last assessment) | Negative | 74(62.2%) | | | 45(37.8%) | | 1.01 | >0.99 |
|  | Positive | 62(62%) | | | 38(38%) | |  |  |
| del(11q) (last assessment) | Negative | 116(63.7%) | | | 66(36.3%) | | 1.23 | 0.43 |
|  | Positive | 21(55.3%) | | | 17(44.7%) | |  |  |
| trisomy 12 (last assessment) | Negative | 120(64.5%) | | | 66(35.5%) | | 1.45 | 0.12 |
|  | Positive | 16(48.5%) | | | 17(51.5%) | |  |  |
| del(17p) (last assessment) | Negative | 132(63.8%) | | | 75(36.2%) | | 1.45 | 0.22 |
|  | Positive | 10(47.6%) | | | 11(52.4%) | |  |  |
| *TP53* mutation status | Mutated | 10(58.8%) | | | 7(41.2%) | | 0.93 | >0.99 |
|  | Unmutated | 92(61.7%) | | | 57(38.3%) | |  |  |
| del(17p) positive and/or  TP53 mutation | YES | 15(53.6%) | | | 13(46.4%) | | 0.83 | 0.58 |
|  | NO | 87(61.3%) | | | 55(38.7%) | |  |  |
| CIRS score | $\leq6$ | 143(70.8%) | | | 59(29.2%) | | **1.62** | **0.004** |
|  | $>6$ | 48(52.7%) | | | 43(47.3%) | |  |  |
| Other respiratory | YES | 15(65.2%) | | | 8(34.8%) | | 1.03 | >0.99 |
|  | NO | 187(64.3%) | | | 104(35.7%) | |  |  |
| Asthma | YES | 5(71.4%) | | | 2(28.6%) | | 1.25 | >0.99 |
|  | NO | 197(64.2%) | | | 110(35.8%) | |  |  |
| COPD | YES | 12(54.5%) | | | 10(45.5%) | | 0.77 | 0.45 |
|  | NO | 190(65.1%) | | | 102(34.9%) | |  |  |
| Cardiac Failure | YES | 3(37.5%) | | | 5(62.5%) | | 0.56 | 0.14 |
|  | NO | 199(65%) | | | 107(35%) | |  |  |
| Arrythmias | YES | 16(66.7%) | | | 8(33.3%) | | 1.08 | 0.98 |
|  | NO | 186(64.1%) | | | 104(35.9%) | |  |  |
| Coronary artery disease | YES | 14(53.8%) | | | 12(46.2%) | | 0.75 | 0.34 |
|  | NO | 188(65.3%) | | | 100(34.7%) | |  |  |
| Other cardiovascular | YES | 21(60%) | | | 14(40%) | | 0.88 | 0.7 |
|  | NO | 181(64.9%) | | | 98(35.1%) | |  |  |
| Hypertension | YES | 100(62.1%) | | | 61(37.9%) | | 0.88 | 0.47 |
|  | NO | 102(66.7%) | | | 51(33.3%) | |  |  |
| Diabetes | YES | 43(66.2%) | | | 22(33.8%) | | 1.07 | 0.84 |
|  | NO | 159(63.9%) | | | 90(36.1%) | |  |  |
| Chronic renal disease | YES | 7(38.9%) | | | 11(61.1%) | | **0.56** | **0.039** |
|  | NO | 195(65.9%) | | | 101(34.1%) | |  |  |
| Other hematological malignancies | YES | 3(75%) | | | 1(25%) | | 1.43 | >0.99 |
|  | NO | 199(64.2%) | | | 111(35.8%) | |  |  |
| Other non-hematological malignancies (excluding skin) | YES | 18(58.1%) | | | 13(41.9%) | | 0.84 | 0.57 |
|  | NO | 184(65%) | | | 99(35%) | |  |  |
| Obesity (BMI>30) | YES | 34(69.4%) | | | 15(30.6%) | | 1.14 | 0.69 |
|  | NO | 167(65.2%) | | | 89(34.8%) | |  |  |
| Smoking | Current smoker | 8(61.5%) | | | 5(38.5%) | |  | 0.07 |
|  | Ex-smoker | 47(55.3%) | | | 38(44.7%) | |  |  |
|  | Never | 127(69.4%) | | | 56(30.6%) | |  |  |
| Hypogammaglobulinemia  (IgG <550 mg/dL) | Present | 73(58.4%) | | | 52(41.6%) | | 0.75 | 0.1 |
|  | Absent | 89(69%) | | | 40(31%) | |  |  |
| CLL treatment status | Treated | 86(51.2%) | | | 82(48.8%) | | **0.42** | **<0.001** |
|  | Untreated | 117(79.6%) | | | 30(20.4%) | |  |  |
| CLL treatment status at the time of COVID-19 | Treated | 46(48.4%) | | | 49(51.6%) | | **0.55** | **<0.001** |
|  | Untreated | 157(71.4%) | | | 63(28.6%) | |  |  |
| Treated in last 12 months | YES | 71(50.7%) | | | 69(49.3%) | | **0.68** | **0.023** |
|  | NO | 62(66.7%) | | | 31(33.3%) | |  |  |
| Antiviral | YES | 89(64.5%) | | | 49(35.5%) | | 0.81 | 0.28 |
|  | NO | 106(71.1%) | | | 43(28.9%) | |  |  |
| Hydroxycloroquine or similar | YES | 85(67.5%) | | | 41(32.5%) | | 0.99 | >0.99 |
|  | NO | 108(67.9%) | | | 51(32.1%) | |  |  |
| Azithromycin | YES | 88(70.4%) | | | 37(29.6%) | | 1.13 | 0.57 |
|  | NO | 103(66.5%) | | | 52(33.5%) | |  |  |
| Steroids | YES | 157(66%) | | | 81(34%) | | 1.01 | >0.99 |
|  | NO | 37(66.1%) | | | 19(33.9%) | |  |  |
| Anti-IL6/IL6R | YES | 41(67.2%) | | | 20(32.8%) | | 0.97 | >0.99 |
|  | NO | 153(66.2%) | | | 78(33.8%) | |  |  |
| **Multivariate analysis** | | | | | | | | |
| **Risk factor** | | | **OR** | **95% CI** | | **p-value** | | |
| Age (years) | | | 1.04 | 1.01 – 1.06 | | **0.002** | | |
| CLL treatment status (Treated vs Untreated) | | | 3.74 | 2.26 – 6.3 | | **<0.001** | | |

*Mutated: <98% germline identity, **Unmutated: ≥98% germline identity

IGHV: immunoglobulin heavy variable, CIRS: Cumulative illness rating scale, COPD: chronic obstructive pulmonary disease, IL6: Interleukin 6, IL6R: Interleukin 6 Receptor,

RR: Relative risk of death

**Figure 1. Number of COVID-19 cases per month.**


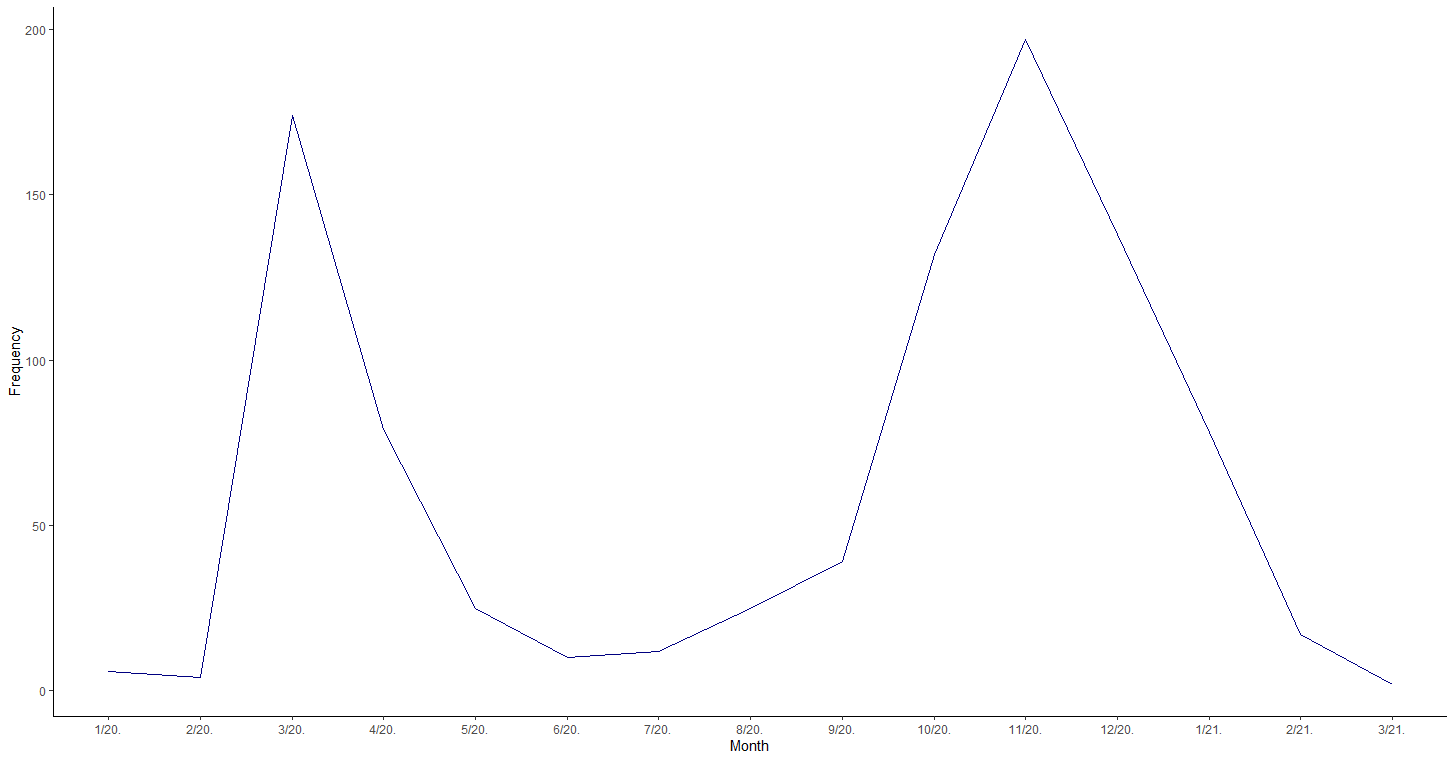


**Figure 2. Overall survival probability for all patients.**


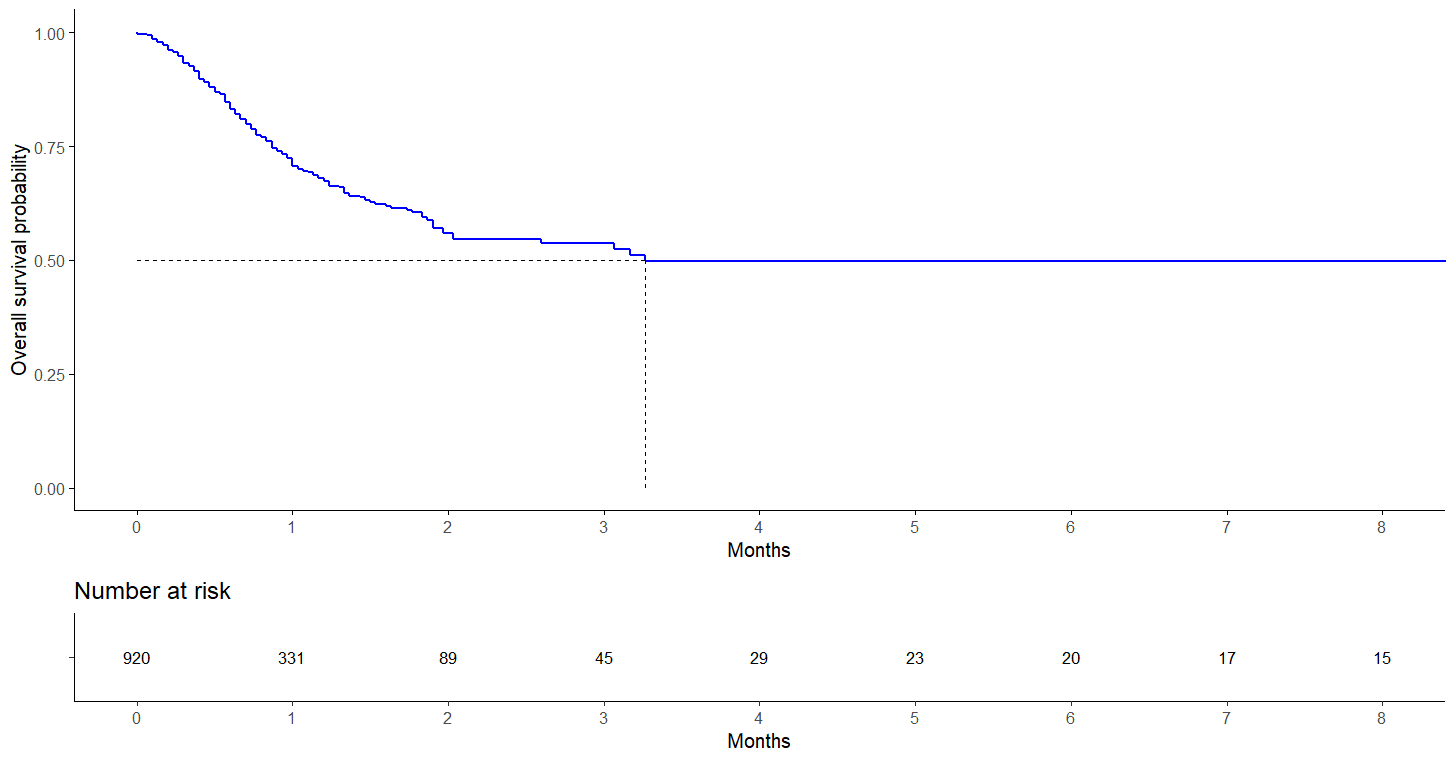


**Figure 3. Overall survival probability for patients with severe COVID-19.**


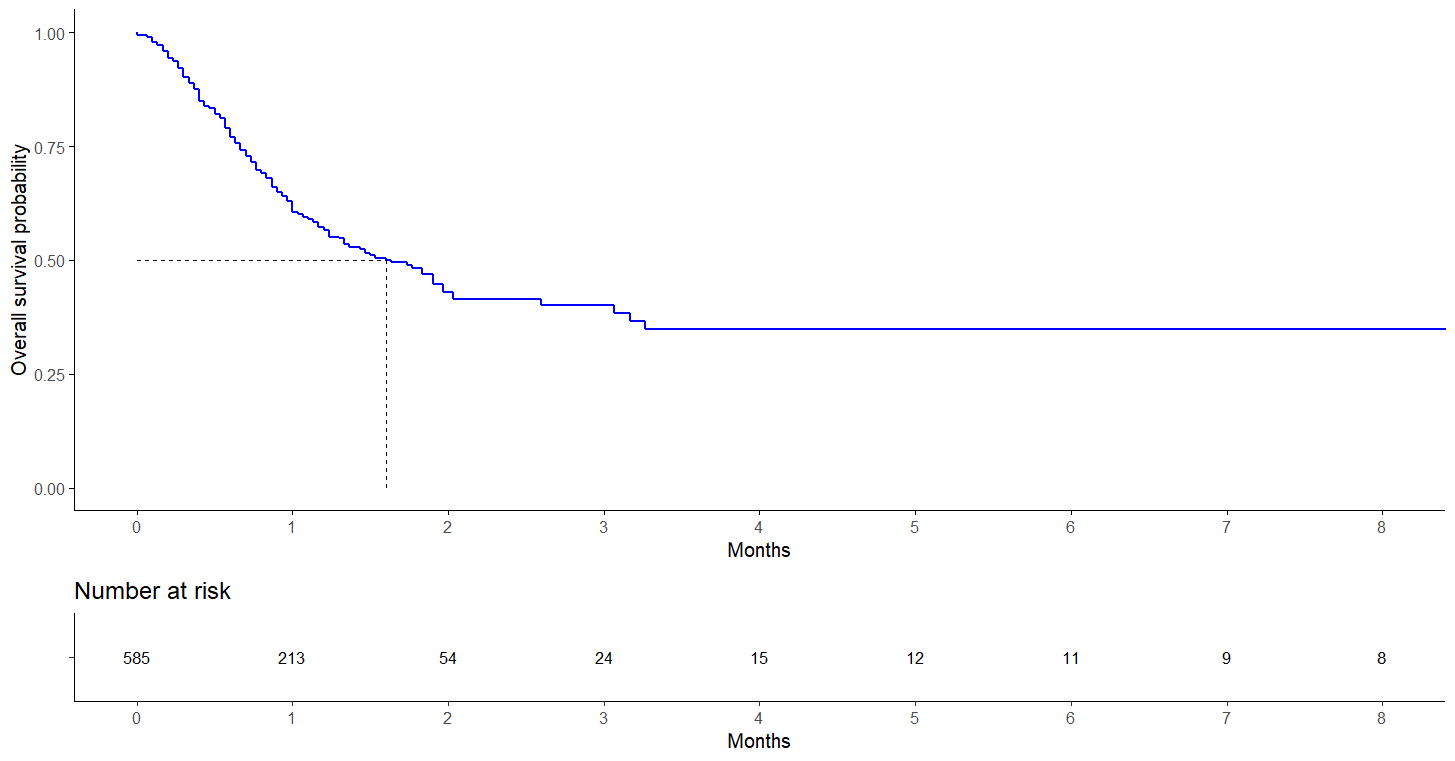


**Figure 4. Overall survival in patients with severe COVID-19 according to treatment: comparisons between BTKi (At time of COVID-19), Venetoclax (At time of COVID-19), Anti-CD20 in the last 12 months and Untreated.**


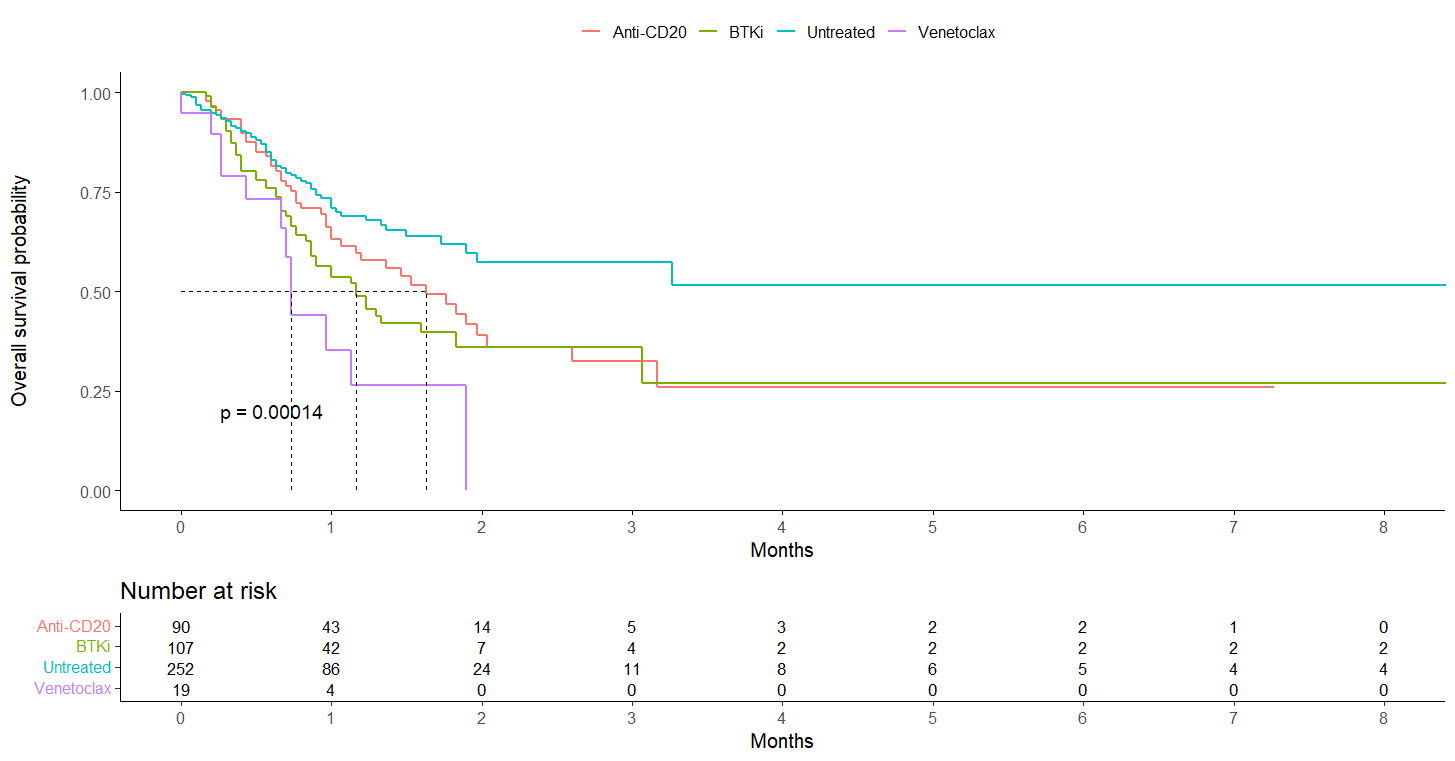

Supplement: Supplementary file 1 — Supplemental information [file 41375_2021_1450_MOESM1_ESM.docx]
